# Threading the Needle: Intrapelvic Displacement of a Femoral Neck Fracture through the Obturator Foramen

**DOI:** 10.1155/2018/2506187

**Published:** 2018-04-01

**Authors:** Gautham Prabhakar, Nicholas Kusnezov, Nicholas Rensing, Amr Abdelgawad

**Affiliations:** Department of Orthopaedic Surgery, Texas Tech University Health Sciences Center, El Paso, TX, USA

## Abstract

Despite timely and appropriate management, displaced femoral neck fractures are often devastating injuries for the young patient. The risk of negative sequelae is further amplified with increasing displacement and vertical fracture patterns. Open anatomic reduction with rigid internal fixation is essential to maximize the healing potential in displaced fractures of the femoral neck. Successful primary osteosynthesis of significantly displaced femoral neck fractures in the young patient has been reported in the literature. We present a unique case of open reduction and internal fixation of a high-energy femoral neck fracture with extrusion of the head through the obturator foramen into the pelvis without associated acetabular or pelvic injury.

## 1. Introduction

Displaced femoral neck fractures are potentially devastating injuries in the young patient [1]. Despite appropriate and timely management, a significant number of these injuries regardless go on to avascular necrosis (AVN), fixation failure, and nonunion with rates ranging from 10 to 45% [2–4]. This is attributable to the tenuous blood supply to the femoral head, which may be disrupted even in nondisplaced fractures [5, 6].

Increasing displacement and vertical nature of the fracture convey an increased risk of complications following fixation [7]. Therefore, for displaced fractures, open anatomic reduction with internal fixation is essential to maximize the healing potential [8–10].

Successful primary osteosynthesis of significantly displaced femoral neck fractures in the young patient has been reported in the literature [11–13]. Previous reports of intrapelvic intrusion of the femoral head following femoral neck fractures have further been described in the setting of pelvic [12] or central acetabular fracture [13]. We present a unique case of open reduction and internal fixation of a high-energy femoral neck fracture with extrusion of the head through the obturator foramen into the pelvis without associated acetabular or pelvic injury.

## 2. Case Presentation

An 23-year-old female polytrauma presented to our academic level 1 trauma center following an unrestrained motor vehicle rollover. On arrival, the patient was hemodynamically stable, alert, and cooperative. Primary survey demonstrated obvious deformities of the right brachium and right thigh as well as a painful, externally rotated left lower extremity. The right brachium was open, but all extremities were neurovascularly intact.

**Figure 1:**
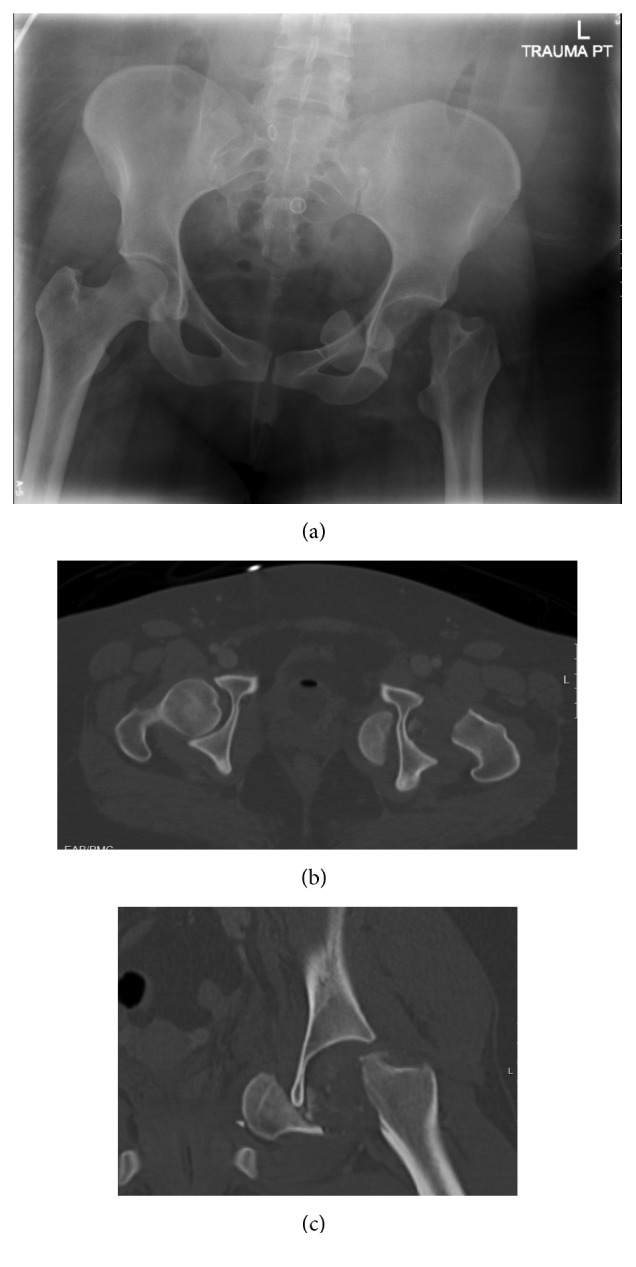
(a) Anteroposterior radiograph demonstrating complete medially displaced left femoral neck fracture. (b, c) Computed tomography (CT) demonstrating the displacement of femoral head in the intrapelvic location, which had extruded through the obturator foramen.

Radiographs demonstrated an open comminuted fracture involving the distal right humerus, a short oblique fracture of the distal right femoral diaphysis, and a complete medially displaced left femoral neck fracture (Figure 1(a)). Computed tomography demonstrated the intrapelvic location of the femoral head, which had extruded through the obturator foramen (Figures 1(b) and 1(c)).

The patient was taken back urgently for operative fixation. Given her age and acuity of the injury, the decision was made to attempt open reduction and internal fixation of the femoral head.

The patient was placed supine on a fracture table and a Smith–Peterson approach was utilized, similar to that for a periacetabular osteotomy. We began with an osteotomy of the anterior superior iliac spine and reflected the sartorius distally. The underlying rectus femoris conjoint tendon was subsequently taken down, exposing the disrupted anterior capsule and fracture site. We then proceeded with recovery of the head, which had displaced into the pelvis and was not retrievable through this approach. The interval between the hip abductors and abdominal musculature was identified and carried down to the iliac crest where the iliacus was released from the iliac bone. The hip was flexed in order to relax the iliopsoas tendon. A Hohmann retractor was then placed over the pubic crest medial to the protuberance. A cup elevator was introduced into the pelvis deep to the iliacus muscle in order to push the femoral head from inside the pelvis. On the other side, the iliocapsularis muscle was released from the hip capsule and the capsule was incised in line with the neck. A combination of pushing the head from inside the pelvis by the cup elevator and pulling the head from the capsule allowed us to retrieve the displaced femoral head.

**Figure 2:**
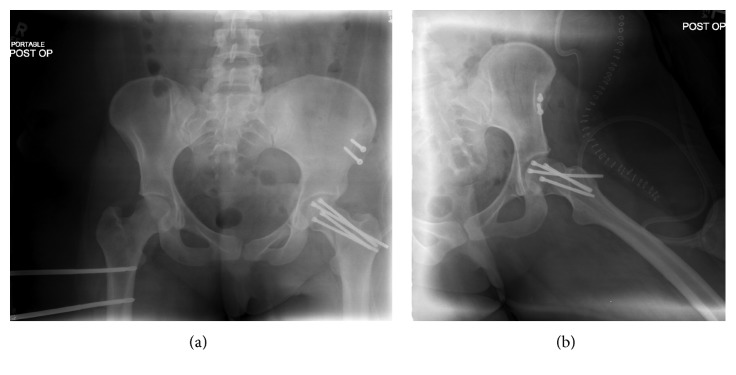
Anteroposterior (a) and lateral (b) radiographs taken immediately postoperatively.

The fracture site was debrided, and the femoral head was replaced. Anatomic reduction was obtained under direct visualization after which provisional fixation was attained with smooth 2.0 mm Kirschner wires. The neck was then definitively fixed with four 6.0 mm partially threaded cannulated headless compression screws (Figures 2(a) and 2(b)). The osteotomy was repaired with two 3.5 mm lag screws. External fixation of the right diaphyseal femur fracture was subsequently performed in the same setting, followed by debridement and irrigation and external fixation of the open left humerus fracture.

**Figure 3:**
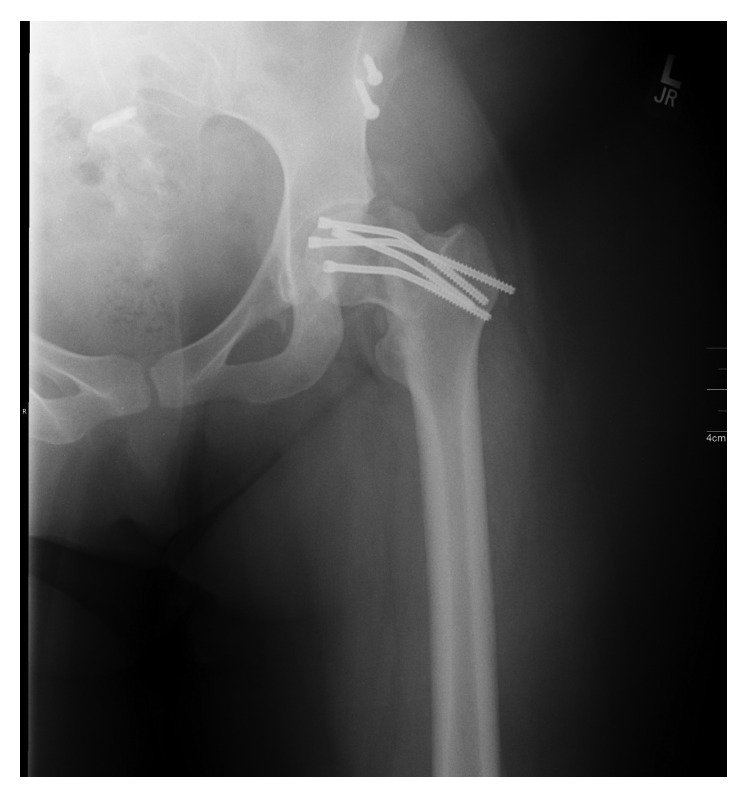
Anteroposterior radiograph showing bending of screws and hardware failure.

The patient was admitted postoperatively, during which she underwent definitive fixation of the right femur on day 2 and open right humerus fracture on day 4. She was discharged home on postoperative day 7. She was maintained non-weight-bearing within a wheelchair for four weeks and subsequently gradually advanced back to full weight-bearing by eight weeks. Radiographs at one-month follow-up demonstrated minimal interval healing though the hardware remained in place without evidence of failure or migration. The patient continued to experience pain with increasing ambulation, and serial radiographs obtained at three and four months demonstrated gradual hardware failure and displacement of the fracture despite appearance of callus and no evidence of AVN (Figure 3). The patient's obesity and noncompliance with weight-bearing likely contributed to a certain degree to the failure of the construct, thus requiring subsequent total hip arthroplasty.

## 3. Discussion

Open reduction and internal fixation (ORIF) should be attempted in the physiologically young patient with a displaced femoral neck fracture. While the risk of nonunion and AVN are similarly high, especially in cases of significant displacement and devitalization of the femoral head [14], the alternative is acute hip arthroplasty. However, arthroplasty is not optimal for the younger population given their age and high level of activity. In addition to a displaced femoral neck fracture in a young patient (generally accepted as <65 years), ORIF is also indicated if inability to achieve acceptable closed reduction persists, or in the setting of posterior communication [8, 10]. Acute anatomic open reduction with rigid internal fixation provides a means by which to maximize healing potential and serves at least to temporize the patient should they need eventual hip replacement.

When considering fixation for displaced femoral neck fractures, the most widely accepted options are either cannulated screws or a sliding hip screw construct. A number of studies have compared the two constructs [15–17]. Cannulated screws require less soft tissue dissection and disruption of the blood supply and necessitate less removal of bone in young patients, effectively preserving more viable bone and optimizing vascularity [4, 18]. Sliding hip screws, while stronger, require more extensive dissection and removal of more bone, especially in young individuals. Cannulated screws require a parallel positioning, which is critical, as this positioning induces compressive forces that stimulate healing [1, 4, 8]. Sliding hip screws may be beneficial in providing intraoperative compression and overcoming the complication associated with the lack of parallel positioning when utilizing cannulated screws. However, poor fixation and loss of reduction may occur as a result of unsatisfactory control of the proximal fragment during insertion of the lag screw [16]. In a prospective randomized controlled trial comparing cannulated screws versus dynamic hip screw, Watson et al. found no difference in union rate, osteonecrosis, or functional outcome [15]. Given the young age of our patient and excellent bone quality, we elected to optimize the amount of viable bone, minimize further soft tissue damage, and thus decided upon cannulated screw fixation. Furthermore, we utilized antegrade headless screws through an anterior surgical hip dislocation. This provided us with maximal appreciation of the reduction and optimal compression across the fracture. While retrograde screws or a sliding hip screw construct is certainly an option and would be admittedly easier to extract in the setting of a total hip arthroplasty, in order to reduce the substantially displaced fracture, we were forced to perform a surgical hip dislocation. In this setting, we felt the fixation and compression provided by antegrade screws were superior to what we could have accomplished with retrograde screws. Currently, there is no literature in favor of either technique over the other.

Previous studies have reported mixed results following internal fixation of significantly displaced femoral neck fractures [18–20]. Meinhard et al. report a case of intrapelvic dislocation of the femoral head through a central acetabular fracture in a 27-year-old policeman following high-energy motorcycle collision [13]. The authors performed a posterolateral approach and were able to deliver the head and neck through the acetabular fracture. The femoral neck fracture was fixed with four partially threaded lag screws. At two-year follow-up, the patient had returned to full activities with no pain, and radiographs demonstrated preserved joint space with no evidence of AVN. While these authors were successful in head retrieval through a posterolateral approach, supine positioning with an anterior approach leaves the surgeon with the option for an ilioinguinal approach (as in our case).

**Figure 4:**
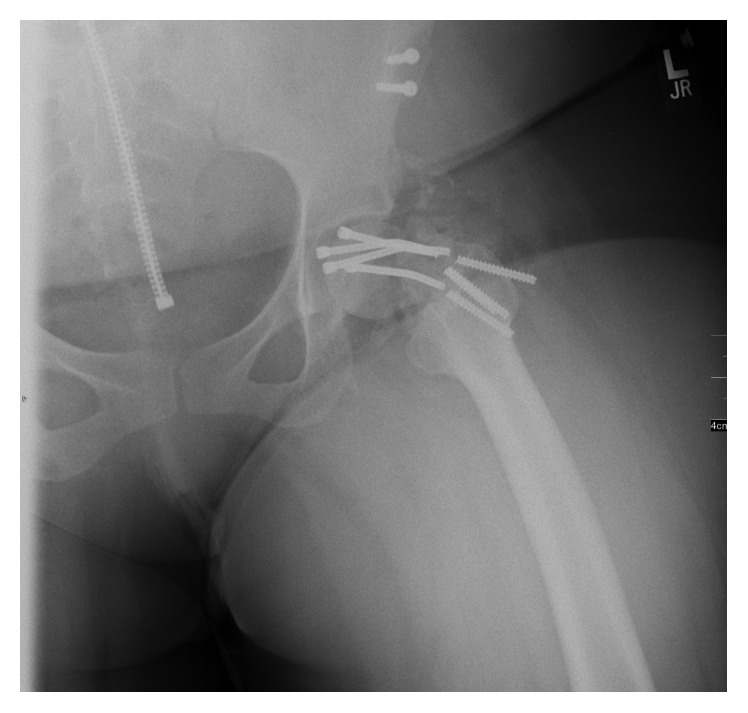
Anteroposterior radiograph demonstrating hardware fatigue and breaking of screws.

Baba et al. report a case of intrapelvic intrusion of the femoral head following femoral neck and pelvic fracture in a 25-year-old male [12]. The authors performed rigid internal fixation following open anterior retrieval of the significantly displaced fragment but reported subsequent AVN of the femoral head as well as heterotopic ossification and ankylosis of the hip. The patient, however, remained asymptomatic despite limited range of motion at five years postoperatively. Therefore, even in the setting of AVN, patients may not require conversion to hip arthroplasty for some time. This supports open reduction and internal fixation as a viable temporizing measure. The patient in our case showed some improvement following open reduction and internal fixation at short-term follow-up and was able to return to weight-bearing. However, the fracture ultimately went on to nonunion with subsequent hardware fatigue and eventual failure (Figure 4). Her obese weight of 100 kg may have increased the shear forces across the fracture site and ultimately contributed to the premature failure of the construct. As a result, the patient was temporized but ultimately converted to total hip arthroplasty nearly 12 months after initial fixation due to symptomatic nonunion.

Schicho and Riepl report the case of a 33-year-old male with a femoral head dislocation into the scrotum following a three-part trochanteric fracture [11]. The fragment was retrieved through a scrotal incision, and the authors performed open reduction with internal fixation with a proximal femoral locking plate. At final 14-month follow-up, computed tomography demonstrated healing of the fracture with no evidence of AVN. Though the fracture in this instance was more basicervical in nature, even in cases where the main vascular supply is compromised, collateral circulation and revascularization can rarely maintain the viability of the femoral head and neck [3].

In our case, anatomic reduction was obtained. Precision and method of reduction have been found to have the most significant influence on healing [1]. Therefore, regardless of the high likelihood of AVN in such cases as these, the authors recommend retrieval of the femoral head and anatomic reduction with rigid internal fixation to maximize healing potential. Should the patient go on to AVN or nonunion as in this case, arthroplasty remains an option. Salvage arthroplasty following failure of internal fixation has generally been found to yield comparably favorable results to primary arthroplasty despite a higher risk of complications such as infection and dislocation [21, 22].
